# Evaluating novel antibodies for cytokine profiling in the guinea pig model

**DOI:** 10.1093/immhor/vlag051

**Published:** 2026-09-22

**Authors:** Lea Maristela, Alyssa Longworth, David F Ackart, Faye Lanni, Joseph Gibb, Ashley Freedman, Olivia R Miller, Ling Cai, Hong Qi, Susan L Baldwin, Sasha E Larsen, Carolina Mehaffy, Marcela Henao-Tamayo, Rhea N Coler, Brendan K Podell

**Affiliations:** Mycobacteria Research Laboratories, Department of Microbiology, Immunology, and Pathology, Colorado State University, Fort Collins, CO, United States; Mycobacteria Research Laboratories, Department of Microbiology, Immunology, and Pathology, Colorado State University, Fort Collins, CO, United States; Mycobacteria Research Laboratories, Department of Microbiology, Immunology, and Pathology, Colorado State University, Fort Collins, CO, United States; Mycobacteria Research Laboratories, Department of Microbiology, Immunology, and Pathology, Colorado State University, Fort Collins, CO, United States; Mycobacteria Research Laboratories, Department of Microbiology, Immunology, and Pathology, Colorado State University, Fort Collins, CO, United States; Mycobacteria Research Laboratories, Department of Microbiology, Immunology, and Pathology, Colorado State University, Fort Collins, CO, United States; Mycobacteria Research Laboratories, Department of Microbiology, Immunology, and Pathology, Colorado State University, Fort Collins, CO, United States; QooLabs, Inc., Carlsbad, CA, United States; QooLabs, Inc., Carlsbad, CA, United States; Phoenix Immune Mechanisms of Protection Against TB (IMPAc-TB) Center, Seattle, WA, United States; Center for Global Infectious Disease Research, Seattle Children’s Research Institute, Seattle, WA, United States; Phoenix Immune Mechanisms of Protection Against TB (IMPAc-TB) Center, Seattle, WA, United States; Center for Global Infectious Disease Research, Seattle Children’s Research Institute, Seattle, WA, United States; Mycobacteria Research Laboratories, Department of Microbiology, Immunology, and Pathology, Colorado State University, Fort Collins, CO, United States; Mycobacteria Research Laboratories, Department of Microbiology, Immunology, and Pathology, Colorado State University, Fort Collins, CO, United States; Phoenix Immune Mechanisms of Protection Against TB (IMPAc-TB) Center, Seattle, WA, United States; Phoenix Immune Mechanisms of Protection Against TB (IMPAc-TB) Center, Seattle, WA, United States; Center for Global Infectious Disease Research, Seattle Children’s Research Institute, Seattle, WA, United States; Mycobacteria Research Laboratories, Department of Microbiology, Immunology, and Pathology, Colorado State University, Fort Collins, CO, United States; Phoenix Immune Mechanisms of Protection Against TB (IMPAc-TB) Center, Seattle, WA, United States

**Keywords:** antibodies, cytokine, guinea pig, tuberculosis

## Abstract

The guinea pig model of *Mycobacterium tuberculosis* (*Mtb*) infection offers distinct advantages compared to conventional mouse models. Namely, the guinea pig model develops circumscribed granulomas with central necrosis which most closely resembles that of human granulomas. However, a limitation of this model is the lack of available reagents to characterize the *Mtb* immune response. To better leverage this representative model, a set of recombinant rabbit monoclonal antibodies targeting guinea pig cytokines were generated and validated. Using established stimulation conditions for primary guinea pig cells capable of producing specific cytokine profiles, the reactivity and specificity of these antibodies to their biological target were evaluated. These antibodies were then evaluated individually for direct ELISA and immunohistochemistry or part of a pair for sandwich ELISA and ELISpot techniques. Utilized in a low-dose aerosol model of *Mtb* in the guinea pig, the utility of these antibodies to detect specific immune cytokines generated in response to infection is demonstrated. Collectively, these antibodies will provide a more comprehensive understanding of immune kinetics, vaccine response, and comparative immunology across the guinea pig model of tuberculosis and other infectious diseases.

## Introduction

Due to the lack of immunological reagents, the guinea pig model has fallen behind other rodent models of infectious disease.^1^ The obstacles faced in characterizing the immune response in guinea pigs has elevated the use of other in vivo models (ie mouse), as immunological reagents are readily available. However, these models are often less translationally relevant to human disease than the guinea pig.

The relevance of the guinea pig model in studying human diseases are due to inherent similarities in immune response and pathophysiology. Guinea pig models have been developed to study various pulmonary, sexually transmitted, gastrointestinal, ocular, and aural infectious diseases.^2^ Guinea pigs are often used in evaluating vaccines against genital herpes^3^ and Lassa fever.^4^ Due to the similarities in reproductive anatomy and development, studies of congenital diseases caused by cytomegalovirus,^5^  *Chlamydia*,^6^ and *Treponema pallidum*^7^ have been conducted with the guinea pig model. Guinea pig models have also proven useful in studying reemerging diseases including Legionnaires’ disease,^8^ shigellosis,^9^ Zika virus disease,^10^ and Ebola hemorrhagic fever.^11^ Though the guinea pig model is highly versatile, the limited availability of immunological reagents has hindered further research using this model.

The guinea pig model is especially important in the study of tuberculosis (TB) because guinea pigs are highly susceptible to infection with *Mycobacterium tuberculosis* (*Mtb*) and recapitulate the characteristics of human infection.^12^ They develop clinical signs, such as weight loss, and characteristic pathology including circumscribed granulomas with central necrosis analogous to humans.^13^^,^^14^ Thus, guinea pigs have been used as a stringent model for evaluating new and established vaccines and treatments against TB.^13^^,^^15–24^ Guinea pigs are also an invaluable tool for studies in relation to latent TB as they can develop a delayed-type hypersensitivity reaction to the tuberculin skin test, one of the standard diagnostic tests for TB.^25^^,^^26^ It is evident that the guinea pig model can provide further insight into the TB disease progression and host immune response that is lacking in other small animal models of TB disease. Hence, the demand for guinea pig immunological reagents is imperative.

A common issue faced by researchers is the reproducibility of data from using inconsistent antibodies that lack thorough validation.^27^ Monoclonal recombinant antibodies are highly specific, making them useful for detecting their targets in many different applications. Further, these antibodies are made from cloned DNA sequences, resulting in increased consistency and reduced lot-to-lot variability.^28^ In this study, we show thorough characterization and validation of novel monoclonal recombinant antibodies generated for detection of guinea pig cytokines. These antibodies were used for immunological assays and applied to the guinea pig model of TB to show their validity.

## Materials and methods

### Monoclonal antibodies

The production of anti-guinea pig rabbit monoclonal antibodies (mAbs) was performed by QooLabs, Inc. (Carlsbad, CA, USA) using their proprietary QMAb (Quick Monoclonal Antibody from Blood) platform. In brief, QMAb is a platform technology that enables the cloning of antibody light and heavy chains coding DNA from B cells obtained from peripheral blood of antigen-immunized rabbits. Cloned full-length coding sequences (Table 1) were expressed in Expi293 (Thermo Fisher) cell expression systems to generate recombinant immunogens. After immunization, cells producing antibodies specific to the immunogen were isolated from rabbit blood for cloning of IgG light and heavy chain sequences, followed by recombinant antibody expression in Expi293 cells. The recombinant mAbs were purified from the culture supernatant using protein A affinity chromatography. Antibodies used for in vivo studies were further purified with Sephacryl S-400 size exclusion chromatography in an endotoxin-free system. Select antibodies were biotinylated to be used in paired assays. For this study, 61 antibody clones were generated across 9 targets.

**Table 1:** GenBank accession numbers used to generate recombinant immunogens.

Cytokine	GenBank accession number
IL-2	NM_001172837
IL-4	NM_001257263
IL-6	XM_013152399
IL-10	NM_001260485
IL-12p40	AB025724
IL-13	XM_003464536
IL-17	JQ315117
CCL2	L04985
TNF-α	NM_001173025

### Animals

Rabbit immunization was performed at the Robert Sargeant Animal Facility (Pacific Immunology), OLAW Assurance ID D16-00612 (A4182-01). All procedures using rabbits were performed with Institutional Animal Care and Use Committee (IACUC) approval under protocol 1. Two New Zealand White rabbits were immunized per antigen with 100 µg of antigen combined with complete Freund’s adjuvant for prime immunization and incomplete Freund’s adjuvant for 2 subsequent boost immunizations. Dunkin–Hartley outbred guinea pigs (350 to 400 g) were obtained from Elm Hill Laboratories. For antibody validation and assay development on in vitro–stimulated cells, peripheral blood mononuclear cells (PBMCs) and bone marrow were collected from guinea pigs at age <4 months. For in vivo validation, guinea pigs were exposed to a low-dose aerosol of *Mtb* Erdman strain as previously described.^29^ In brief, exposure to a low-dose aerosol of *Mtb* was performed using the Glas-Col whole-body aerosol exposure device. Saline containing 5 mL of 2 × 10^6^ CFU of *Mtb* Erdman strain (BEI Resources, #NR-15404) was added to the nebulizer device. Guinea pigs were exposed to the nebulization for 3 minutes, targeting an exposure dose of 20 to 50 CFU. Confirmation of the exposure dose at 24 hours postexposure was performed, yielding a mean of 24 CFU isolated from the lung. All procedures involving *Mtb* were performed in the Biosafety Level 3 (BSL3) laboratory facility at Colorado State University by trained laboratory staff approved to work under BSL3 conditions and following approved project protocols by the institutional biosafety committee. Animals were euthanized on day 28 postinfection. All procedures using guinea pigs were conducted at Colorado State University (OLAW D16-00345 [A3572-01]) with IACUC approval under protocol numbers 4598 and 3853.

### Recombinant antigens and antigen-specific cell isolation from rabbits

Recombinant antigens were expressed in Expi293F cells (Thermo Scientific, #A14527) as secreted proteins with a 6X His tag at the C-terminus. Cells were grown in Expi293 Expression Medium (Thermo Scientific, #A1435101) per the manufacturer’s guidelines. Each protein was affinity purified from cell culture supernatant using His60 Ni Superflow Resin (Takara Bio, #635661). Antigen-specific B cells were purified from PBMCs of vaccinated rabbits using proprietary methods established by Qoolabs, Inc. PBMCs were isolated using gradient centrifugation with Ficoll-Paque Plus (Cytiva, #17144003) per the manufacturer’s guidelines.

### Primary guinea pig cell isolation and sample generation

To determine which antibody clones were viable for use in downstream assays, supernatants that produced cytokines of interest to the trialed antibody needed to be generated. Supernatants were generated utilizing isolated guinea pig primary cells including PBMCs and bone marrow–derived macrophages (BMDMs).

PBMCs were isolated from freshly collected whole blood using density gradient centrifugation with Lympholyte Mammal (Cedarlane, #CL5120) as described in the manufacturer’s protocol. To generate supernatants from PBMCs, isolated cells were plated with OptiMEM (Gibco, #31985-070) at 2 million cells per well in a 24-well cell culture plate and stimulated according to desired cytokine target (Table 2). Supernatant was collected and assayed by ELISA.

**Table 2:** Stimulation conditions used to generate each cytokine target ex vivo.

Target	Cell type	Stimulation conditions	References
IL-2, IL-17	PBMCs	5 μg/mL concanavalin A (Invitrogen, #00-4978-93) for 48 hours	^30^ ^,^ ^31^
IL-6, CCL2	BMDMs	100 ng/mL LPS (Invitrogen, #00-4976-93) for 72 hours	^32^ ^,^ ^33^
IL-4, IL-10, IL-13	BMDMs	100 ng/mL LPS and 500 μM prostaglandin E2 (Tocris, #2296) for 48 hours	^34^ ^,^ ^35^
IL-12p40, TNF-α	BMDMs	Bacille Calmette-Guérin (BCG Danish 1331 strain) at a 5:1 multiplicity of infection for 72 hours; 100 ng/mL LPS for 24–72 hours	^36^ ^,^ ^37^

BMDMs were derived from bone marrow progenitor cells as previously described.^38^ Isolated cells were treated with human macrophage colony-stimulating factor (M-CSF) (GeminiBio, #300-161P) every 3 days. On day 7, BMDMs were isolated from the culture and were used immediately in ELISpot assays or cultured with stimuli for supernatant generation. To generate supernatant, cells were plated in OptiMEM serum-free media at 5 × 10^5^ cells per well in a 6-well cell culture plate and stimulated according to desired cytokine target (Table 2). The supernatant was collected and assayed by ELISA.

### Sample generation at necropsy

At the point of necropsy, serum and PBMCs were obtained from blood and cryopreserved for downstream assays. For immunohistochemistry (IHC), lung tissue was collected and immersion fixed in 4% formaldehyde (Electron Microscopy Services, #15714-S) for 48 hours prior to embedding and sectioning. For ELISA, lung tissue was collected into a 15-mL CK50 Precellys tube (Bertin Instruments) and diluted 1:10 with homogenization buffer containing 1× Halt protease inhibitor (Thermo Scientific, #78438) in 1× PBS with 0.05% Tween 20. The tissue was homogenized on a Precellys Evolution Touch homogenizer at 7,200 rpm for 16 seconds. The homogenate was centrifuged at 10,000 × *g* for 5 minutes. Supernatant was collected and filtered with a 0.2-μm filter (Millipore) prior to being assayed.

### Direct ELISA

High-binding 96-well plates (Corning, #3369) were coated with recombinant immunogen, unstimulated supernatant, and cytokine-enriched supernatant generated from cell cultures as described in Table 2. After an 8-hour incubation at 4 °C, plates were blocked with 0.5× casein solution in water (Vector Lab). Unconjugated mAbs (QooLabs) were diluted to 1/32 in blocking buffer and added to the wells; the plate was then incubated for 1 hour at room temperature. HRP-conjugated goat anti-rabbit antibody (Life Tech) was added to each well, and plates were incubated for 1 hour at room temperature. Plates were washed 5 times with PBS–Tween 80 between each step in the procedure. Following the final incubation, 1× Ultra 3,3’,5,5’-tetramethylbenzidine (TMB) (Thermo Scientific, #34028) was added into each well. After 10 minutes, 2 M sulfuric acid was added to the wells to stop the reaction. Plates were analyzed for colorimetric absorbance, measured at 450 nm, using a spectrophotometer. Raw absorbance values were normalized across 3 biological replicates, each including 3 technical replicates, by scaling to minimum and maximum values across all stimulated and unstimulated replicates combined from each antibody clone and respective target.^39^

### Sandwich ELISA

High-binding 96-well plates were coated with 10 µg/mL of unconjugated mAbs. After an 8-hour incubation at 4 °C, plates were blocked with 0.5× casein solution (Vector Laboratories, #SP-5020). Recombinant immunogen, unstimulated supernatant, and cytokine-enriched supernatant were added to the wells and incubated for 2 hours. Biotinylated mAbs (QooLabs), diluted with blocking buffer to 1 µg/mL, were added to the wells. After a 1-hour incubation, HRP-conjugated streptavidin (Thermo Scientific, #N100) was added to each well and plates were again incubated for 1 hour at room temperature. Plates were washed 5 times with PBS–Tween 80 between each step in the procedure. Following the final incubation, TMB was added into each well. After 10 minutes, 2 M sulfuric acid was added to the wells to stop the reaction. Plates were analyzed for colorimetric absorbance, measured at 450 nm, using a spectrophotometer.

### Immunoprecipitation and SDS-PAGE

Immunoprecipitation (IP) was performed to confirm cytokine target specificity of each mAb. A Pierce MS-Compatible Magnetic IP Kit (Thermo Scientific, #90409) was used following the manufacturer’s protocol. IP was performed using a relevant biological sample (cell culture supernatant) plus antibody, recombinant protein sample plus antibody, and a control with only antibody. After the IP process, samples were stripped from the magnetic beads, concentrated in a SpeedVac, and reconstituted in 10 mM ammonium bicarbonate sample buffer. The entire sample from each IP prep was loaded into a NuPAGE 4% to 12% Bis-Tris gel (Thermo Scientific, #NP0321) in MES running buffer. Electrophoresis was performed at 200 V for 35 minutes. Pierce Silver Stain for Mass Spectrometry (Thermo Scientific, #24600) was used to visualize protein bands.

### Chromogenic immunohistochemistry

Slide deparaffinization and all IHC procedures were performed on a Leica Bond RXm automated slide stainer, utilizing standard Leica bulk reagents. Marker detection was carried out with the 3,3’-diaminobenzidine (DAB) BOND Polymer Refine Detection kit (Leica Biosystems, #DS9800), with the following modifications: 2.5% goat serum (Vector Laboratories, #S-1012) replaced the Leica blocking solution, and the ImmPRESS HRP goat anti-rabbit IgG polymer secondary antibody (Vector Laboratories, #MP-7451) replaced the anti-mouse HRP secondary antibody; additionally, freshly prepared 3% hydrogen peroxide and an alternative hematoxylin counterstain (Dako, #CS700) were employed. Heat-induced epitope retrieval was conducted at 95 °C for 20 or 40 minutes, using either citrate buffer (pH 6; Leica Biosystems, #AR9961) or EDTA buffer (pH 9; Leica Biosystems, #AR9640). Endogenous peroxidase activity was quenched with 3% hydrogen peroxide in water (MilliporeSigma, #216763). 2.5% normal goat serum served as a blocking agent and primary antibody diluent. Tissue sections were incubated with primary antibodies (QooLabs), followed by secondary incubation with ImmPRESS HRP polymer-conjugated antibodies. Markers were visualized with DAB chromogen, alongside hematoxylin counterstaining. Slides were dehydrated through graded ethanol solutions, cleared with xylene, and cover-slipped with xylene-based mounting media using the Leica CV5030 automated cover slipper (Leica Biosystems).

### Morpholino knockdown

Translation-blocking morpholino antisense oligonucleotides (Gene Tools) were designed with sequence homology near or overlapping the AUG translational start site of the mRNA of the cytokine targets. On day 0, cells were plated and stimulated as described above in primary cell isolation methods. Morpholinos and polyethylene glycol–based Endo-Porter reagent (Gene Tools) were added into each well for a final concentration of 5 µM and 6 µM for CCL2, TNF-α, and IL-12p40; or 5 µM and 10 µM for IL-6, respectively. Standard control morpholino oligonucleotides (Gene Tools) were used as a nonspecific control. Supernatant was removed from the cells at 24 and 48 hours after stimulation and assayed by ELISA.

### ELISpot

Polyvinylidene difluoride–lined 96-well plates (MilliporeSigma, #MSIPN4W50) were activated with 70% ethanol, prior to coating with unconjugated mAbs. After an 8-hour incubation at 4 °C, plates were washed 5 times with PBS and blocked with Super Block (Thermo Scientific, #37545). Super Block was prepared as per manufacturer’s protocol. Plates were incubated for 2 hours at room temperature. After the incubation, the supernatant was removed from the wells. 1 × 10^5^ cells were plated into each well containing media, with the stimulations outlined in Table 2 or with media alone as a control. Samples were incubated for 48 hours at 37 °C with 5% CO_2_. Plates were then washed 5 times with PBS–0.05% Tween 20. Biotinylated mAbs were added into each well and incubated for 2 hours at room temperature. After washing 5 times with PBS–0.05% Tween 20, HRP-conjugated streptavidin was added to each well for 1 hour at room temperature. Plates were washed 5 times with PBS–0.05% Tween 20 and 5 times with PBS alone prior to adding TMB (Mabtech, #3651-10) for 20 minutes. Plates were washed 10 times with water to stop the reaction and air-dried overnight, before being scanned and analyzed using a CTL Immunospot Plate Imager and Visiopharm image analysis for spot detection and counting.

### Statistical analysis

A min-max normalization was applied to replicate absorbance values.^39^ Significance was determined between the stimulated and unstimulated supernatants using an unpaired t-test or, when necessary, Welch’s t-test. The assay was performed in triplicate with a minimum of 2 technical replicates. For sandwich ELISA data, significance was determined between the stimulated and unstimulated supernatants using an unpaired t-test or, when necessary, Mann–Whitney t-test. The assay was performed in triplicate with a minimum of 2 technical replicates. For statistical analysis of morpholino knockdown efficiency, one-sample t-test was used to compare the mean percent knockdown to zero, where nonspecific morpholino controls did not differ. The assay was performed in duplicate, each with 3 technical replicates. For ELISpot data, count data were log_10_-transformed, and an unpaired t-test, or, when necessary, Welch’s t-test, was used to compare stimulated and unstimulated cell conditions.

## Results

### Evaluation of antibody reactivity

**Figure 1:**
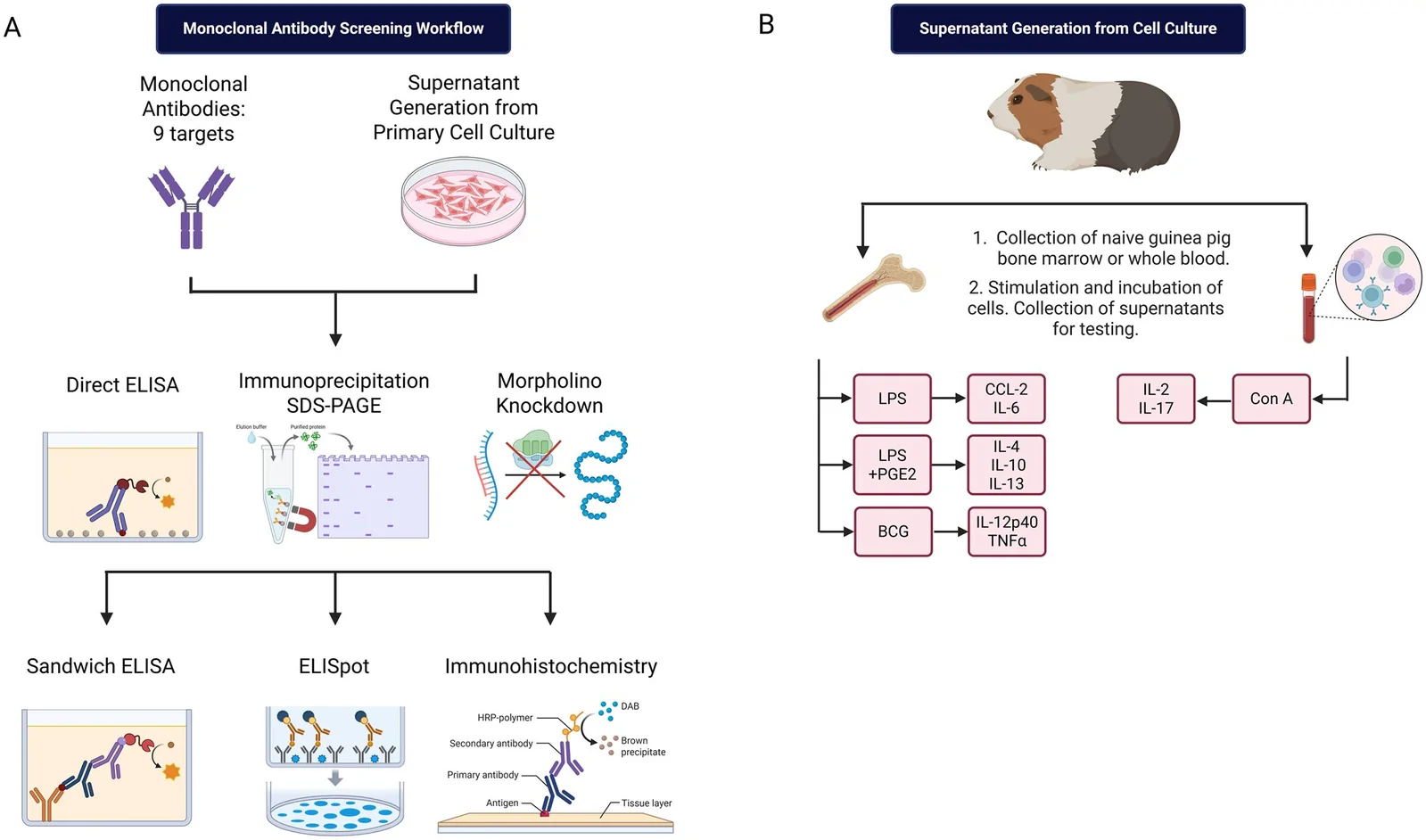
Workflow used to evaluate, validate, and optimize antibodies. (A) Monoclonal antibody screening workflow. (B) Supernatant generation from primary guinea pig cells and stimulants producing specific cytokine profiles. Created in BioRender. Podell, B. (2025), https://BioRender.com/4035r34.

**Figure 2:**
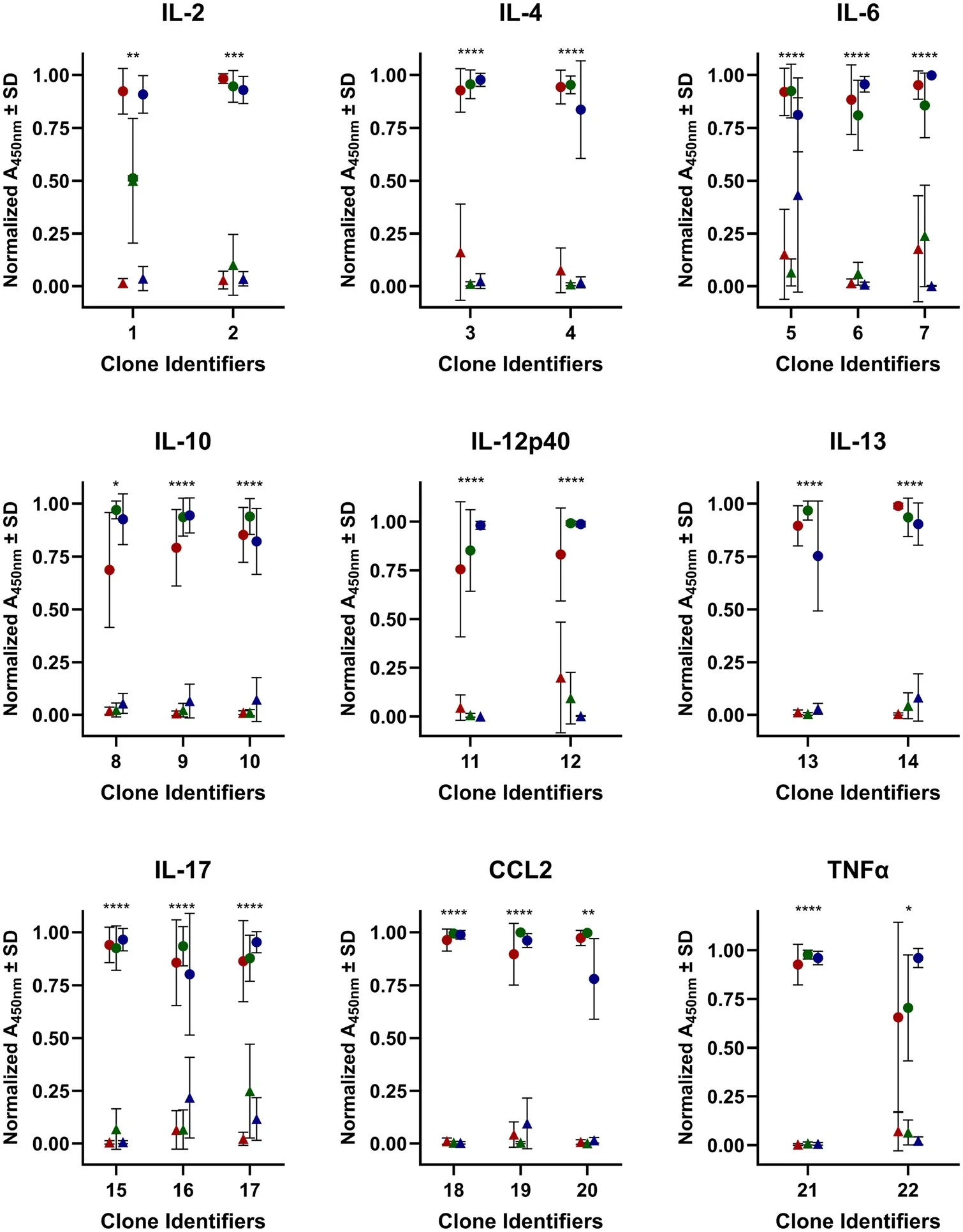
Antibody screening using direct ELISA. Antibodies were tested against supernatants from stimulated cells and unstimulated cells using conditions and cell types described in Table 2. Normalized absorbance values are shown, scaled to the minimum and maximum OD_450_ absorbance values across stimulated (circles) and unstimulated (triangles) conditions for each cytokine target. Each symbol represents the mean of 2 or 3 technical replicates within an experiment. Symbols matched by color represent the outcome of stimulated and unstimulated supernatants of 3 independent biological replicates from supernatant generation through ELISA detection. Raw absorbance values are listed in Table S2. *P < 0.05, **P < 0.01, ***P < 0.001, ****P < 0.0001, unpaired t-test or Welch’s t-test comparing the normalized mean and SD across 3 biological replicates.

The workflow to characterize the reactivity and specificity of each antibody clone is shown schematically in Fig. 1. Initially, direct ELISA was used to evaluate the reactivity of each antibody clone, against supernatants from stimulated cells that produce the intended biological target (Table 2). Of the 61 clones that were trialed using direct ELISA, only 22 clones were found to be reactive to the induced native target in enriched supernatant (Fig. 2). Clone identifiers are detailed in Table S1. Each of the 22 clones found to be reactive demonstrated significantly higher absorbance values in the stimulated cell supernatants compared to unstimulated control supernatants. The means of raw OD_450_ absorbance values are shown in Table S2.

**Figure 3:**
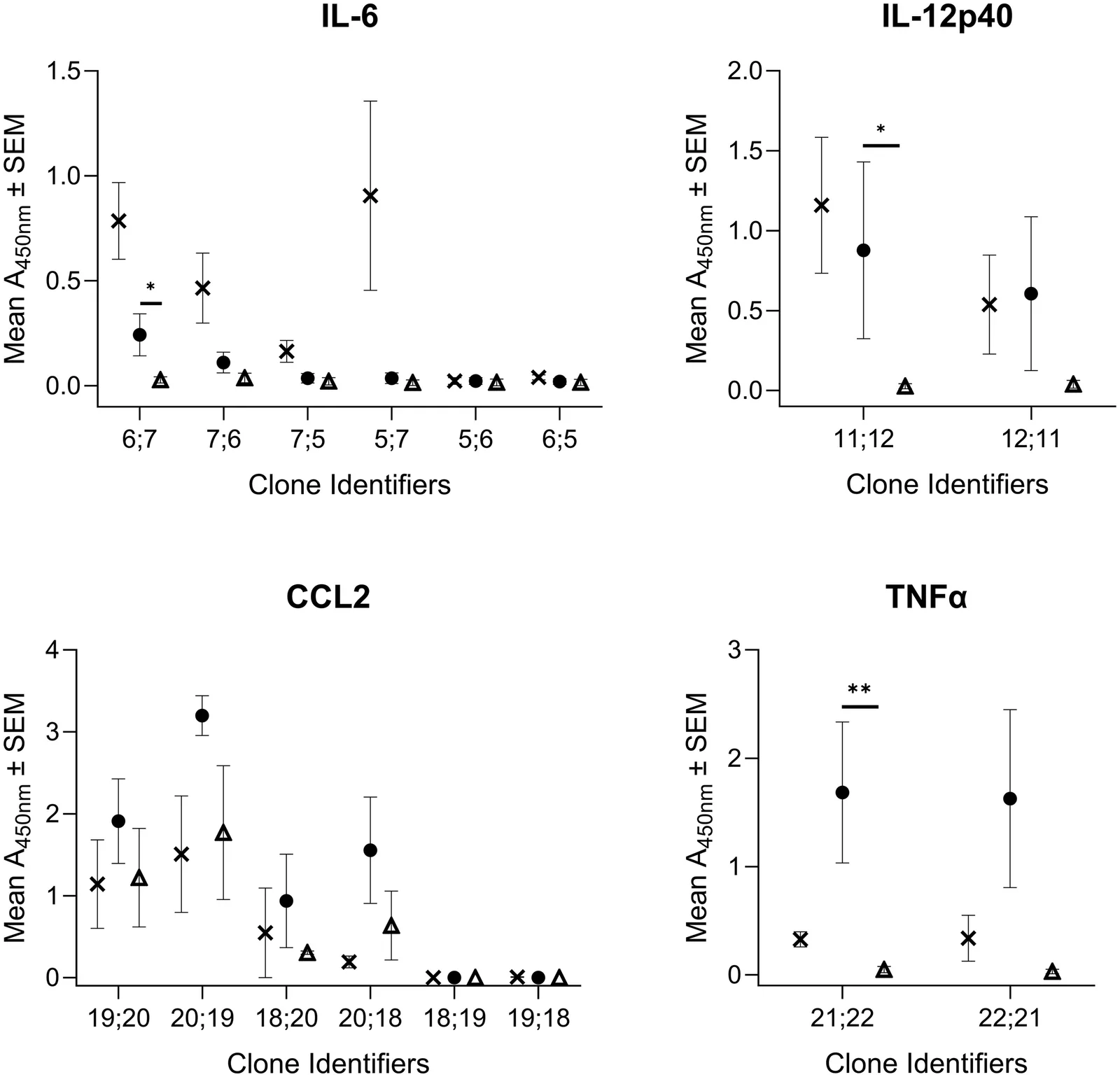
Antibody pair evaluation using sandwich ELISA. Antibodies (capture; detection) were tested against supernatants from stimulated cells and unstimulated BMDMs using conditions described in Table 2. Mean absorbance values are shown for the recombinant immunogen (cross symbols), stimulated supernatant (closed circles), and unstimulated supernatant (open triangles) conditions for each cytokine target. Each symbol represents the mean of 3 biological replicates; each performed with at least 2 technical replicates. *P < 0.01, **P < 0.01, unpaired t-test or Mann–Whitney t-test comparing the mean and SEM of stimulated supernatant to unstimulated supernatant.

A sandwich ELISA method has increased specificity and sensitivity when compared to direct ELISA; thus, the 22 reactive clones were tested as pairs for sandwich ELISA. Pairs were determined by trialing all possible combinations of antibody-specific clones. Detection of the recombinant immunogen and biological target from supernatants, without an increase in background, was used as the primary criterion for choosing the most reactive pair. The performance of each antibody pair targeting IL-6, IL-12p40, CCL2, and TNF-α are shown in Fig. 3. Additionally, working antibody pairs for IL-17 and IL-10 were determined whereas no working pairs were identified for IL-2, IL-4, and IL-13 (data not shown). Based on significantly greater detection in supernatant from stimulated primary cells compared to unstimulated supernatant, antibody pairs for sandwich ELISA were identified for targets CCL2 (clone 19 capture, clone 20 detection), IL-6 (clone 6 capture, clone 7 detection), IL-12p40 (clone 11 capture, clone 12 detection), and TNF-α (clone 21 capture, clone 22 detection).

### Evaluation of antibody specificity

**Figure 4:**
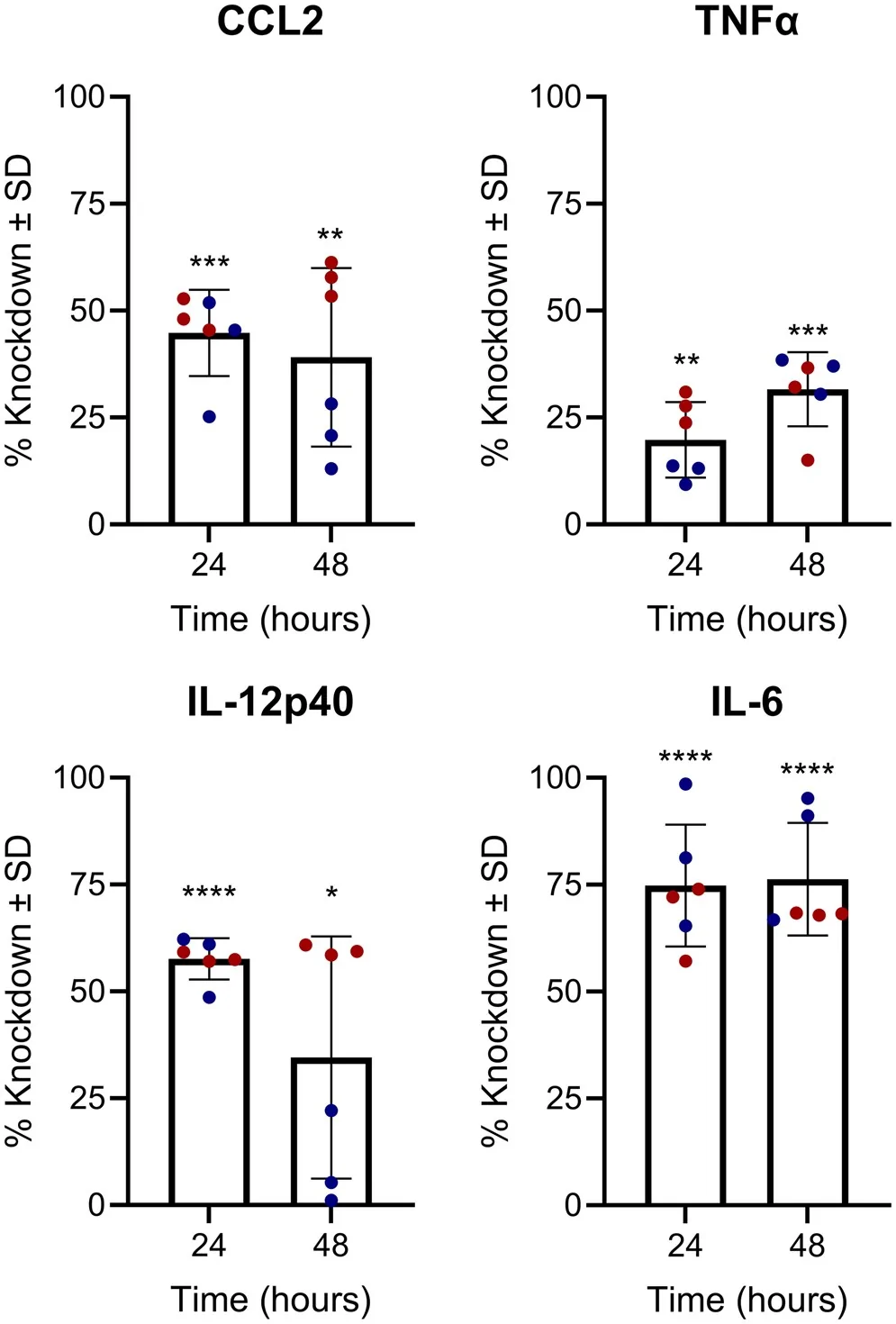
Validation of antibodies. Antibody specificity for targets functional in sandwich ELISA applications, TNF-α (clone 21 capture, clone 22 detection), CCL2 (clone 19 capture, clone 20 detection), IL-12p40 (clone 11 capture, clone 12 detection), and IL-6 (clone 6 capture, clone 7 detection), were confirmed by translation blocking morpholinos. Specific morpholinos were added to BMDMs after addition of stimulation conditions outlined in Table 2 and knockdown expressed as the proportion of reduced target compared to the nonspecific morpholino control. The percent knockdown determined from concentration of cytokine measured at 24 and 48 hours after addition of morpholino is shown across 2 biological replicates of 3 technical replicates each from supernatant generation, in the presence or absence of translation-blocking morpholinos, through ELISA detection. *P < 0.05, **P < 0.01, ***P < 0.001, ****P < 0.0001, one-sample t-test comparing knockdown to zero where control morpholino conditions were not significant.

To determine the specificity of the antibodies, a series of validation steps were conducted. All clones reactive to both enriched supernatant and recombinant immunogen, as determined by direct ELISA (Fig. S1), were subjected to IP targeting the recombinant immunogen and associated enriched supernatant (Fig. S2). All reactive clones precipitated the recombinant immunogen at their respective molecular size. If IP data did not show specificity from supernatant samples based on the band location of the recombinant immunogen, a second confirmatory approach was attempted with morpholinos. Translation-blocking morpholinos (Table S3) were used to prevent production of cytokines and measure the degree of specific protein knockdown using sandwich ELISA. The percent knockdown was calculated by comparing the concentration of the target in wells incubated with targeted morpholino and wells incubated with nonspecific control morpholino. Knockdown by translation blocking of TNF-α, CCL2, IL-12p40, and IL-6 protein were measured by sandwich ELISA (Fig. 4). At 24 hours, a mean reduction of 19.8%, 44.8%, 57.6%, and 74.8% was observed for TNF-α, CCL2, IL-12p40, and IL-6, respectively. At 48 hours, a mean reduction of 31.6%, 39.1%, 34.6%, and 76.3% was observed for TNF-α, CCL2, IL-12p40, and IL-6, respectively. For clones suitable for IL-6 detection by IHC, blocking with recombinant immunogen was performed and further demonstrated specificity based on loss of immunoreactivity (Fig. S3).

### Optimization of antibody pairs for ELISpot

**Figure 5:**
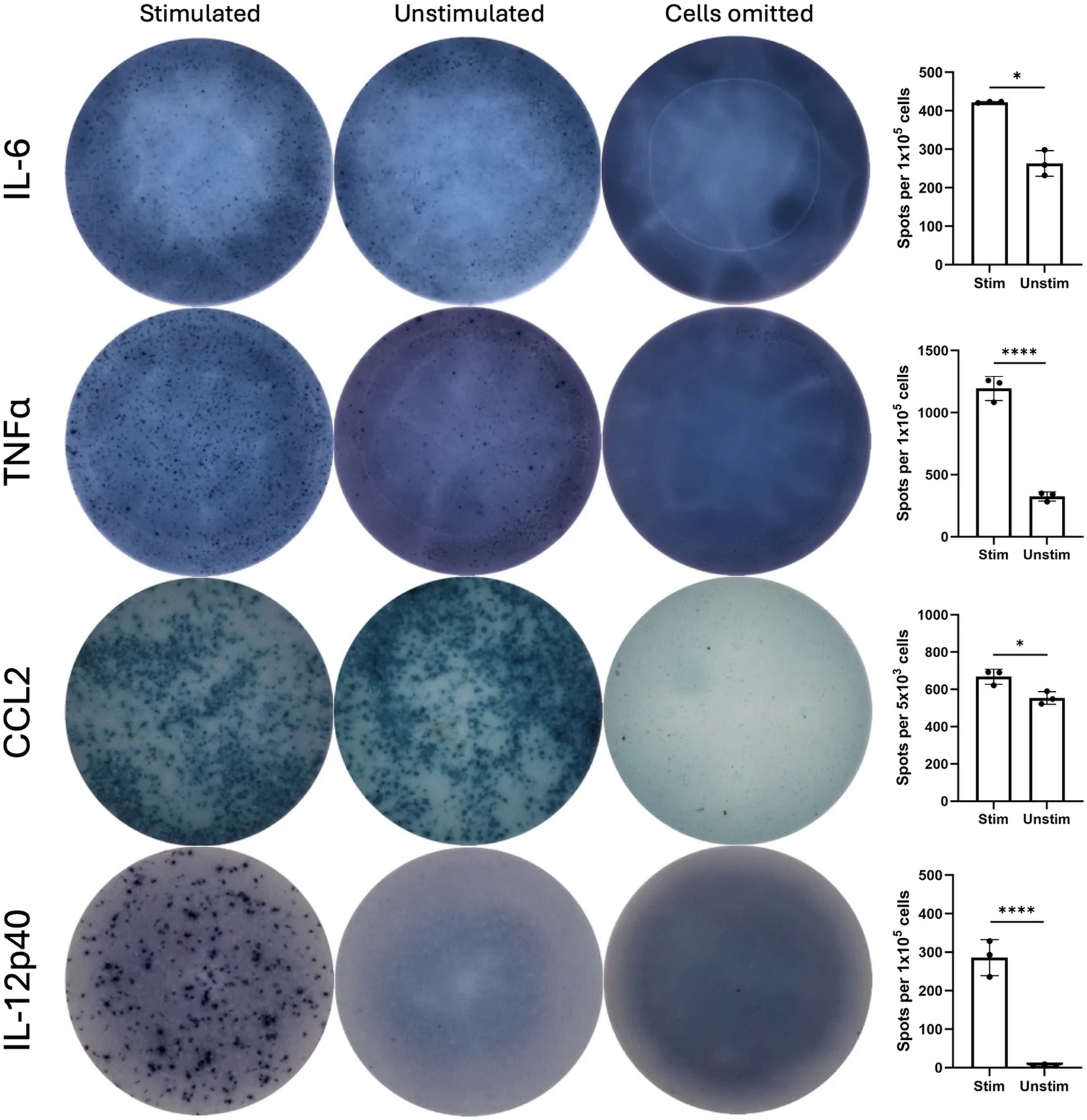
Antibody pair evaluation using ELISpot. Quantification of spot-forming units between unstimulated and stimulated wells for each cytokine target. For IL-6 (clone 6 capture, clone 7 detection) and TNF-α (clone 21 capture, clone 22 detection), images were obtained with 1 × 10^5^ PBMCs/well without stimuli or stimulated with LPS and a well with cells omitted. For CCL2 (clone 19 capture, clone 20 detection), images were obtained with 5 × 10^3^ PBMCs/well without stimuli or stimulated with LPS and a well with cells omitted. For IL-12p40 (clone 11 capture, clone 12 detection), images were obtained with 1 × 10^5^ BMDMs/well without stimuli or stimulated with BCG and a well with cells omitted. *P < 0.05, ****P < 0.0001, unpaired t-test or Welch’s t-test comparing stimulated to unstimulated spot counts.

Antibody pairs, determined by sandwich ELISA, were evaluated for ELISpot. The ability to analyze cytokine production at a single-cell level is invaluable for vaccine and therapeutics research; thus, the optimization of these antibodies for ELISpot was essential. IL-6 and TNF-α pairs were tested against LPS-stimulated and unstimulated guinea pig PBMCs at 1 × 10^5^ cells per well. CCL2 pairs were tested against LPS-stimulated and unstimulated guinea pig BMDMs at 5 × 10^3^ cells per well. IL-12p40 pairs were tested against BCG-stimulated and unstimulated guinea pig BMDMs at 1 × 10^5^ cells per well. Wells with cells omitted were incorporated as a negative control to measure background binding. Stimulated wells developed more spots compared to unstimulated wells, except for CCL2, where spot counts for both conditions were similar (Fig. 5).

### Ex vivo validation

**Figure 6:**
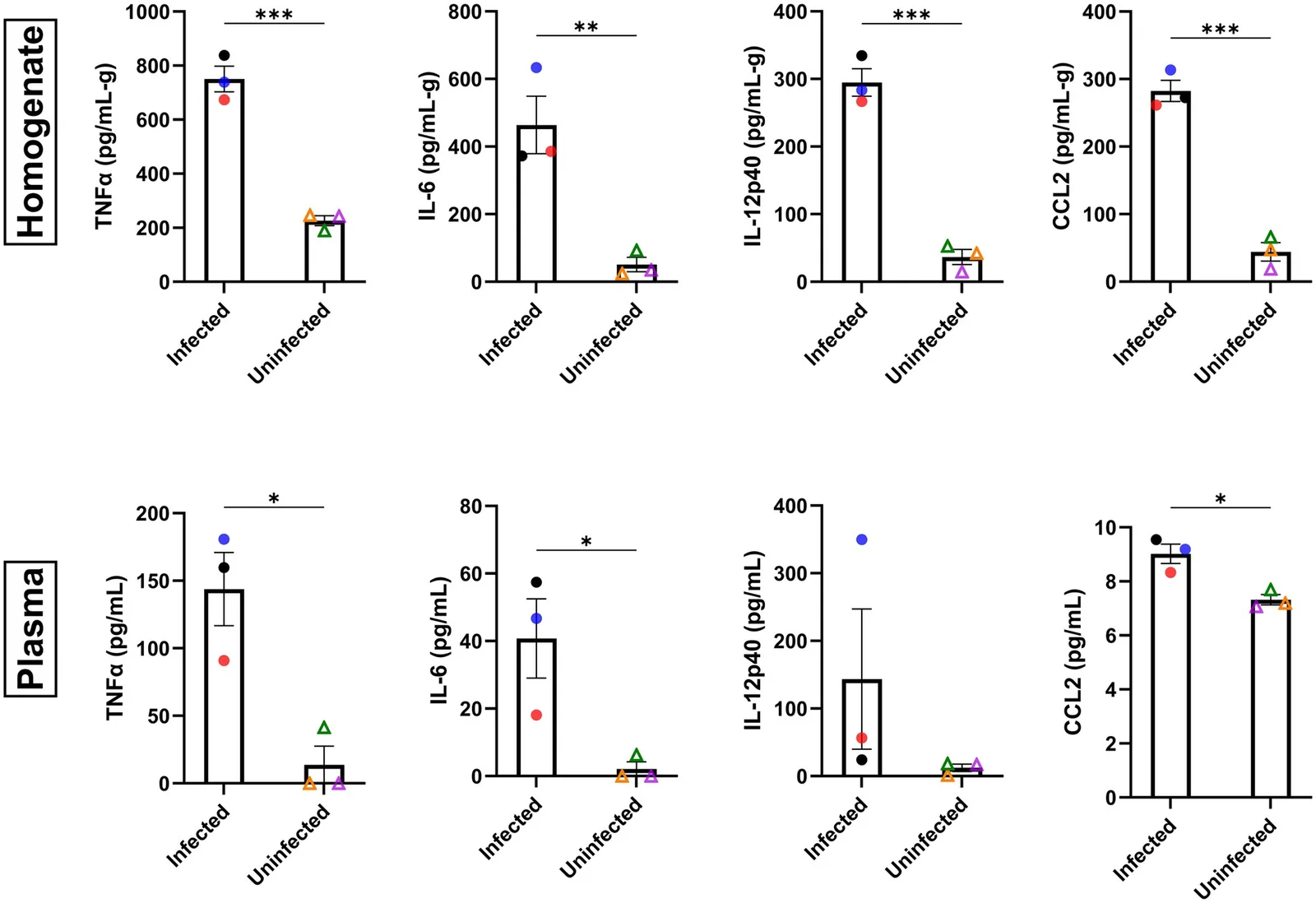
Ex vivo validation using sandwich ELISA. Uninfected or *Mtb*-infected guinea pigs (*n* = 3 per group) were necropsied at day 28 postinfection. At point of necropsy, lungs and plasma were collected from each animal and prepared as described in the Materials and Methods. Concentrations of IL-6 (clone 6 capture, clone 7 detection), IL-12p40 (clone 11 capture, clone 12 detection), TNF-α (clone 21 capture, clone 22 detection), and CCL2 (clone 19 capture, clone 20 detection) are measured as picograms per milliliter-gram of tissue (pg/mL-g) in homogenate or picograms per milliliter (pg/mL) in plasma across infection status. Each animal was performed with 2 technical replicates. Columns represent the mean of infected or uninfected animals and error bars represent SEM. *P < 0.05, **P < 0.01, ***P < 0.001, unpaired t-test.

**Figure 7:**
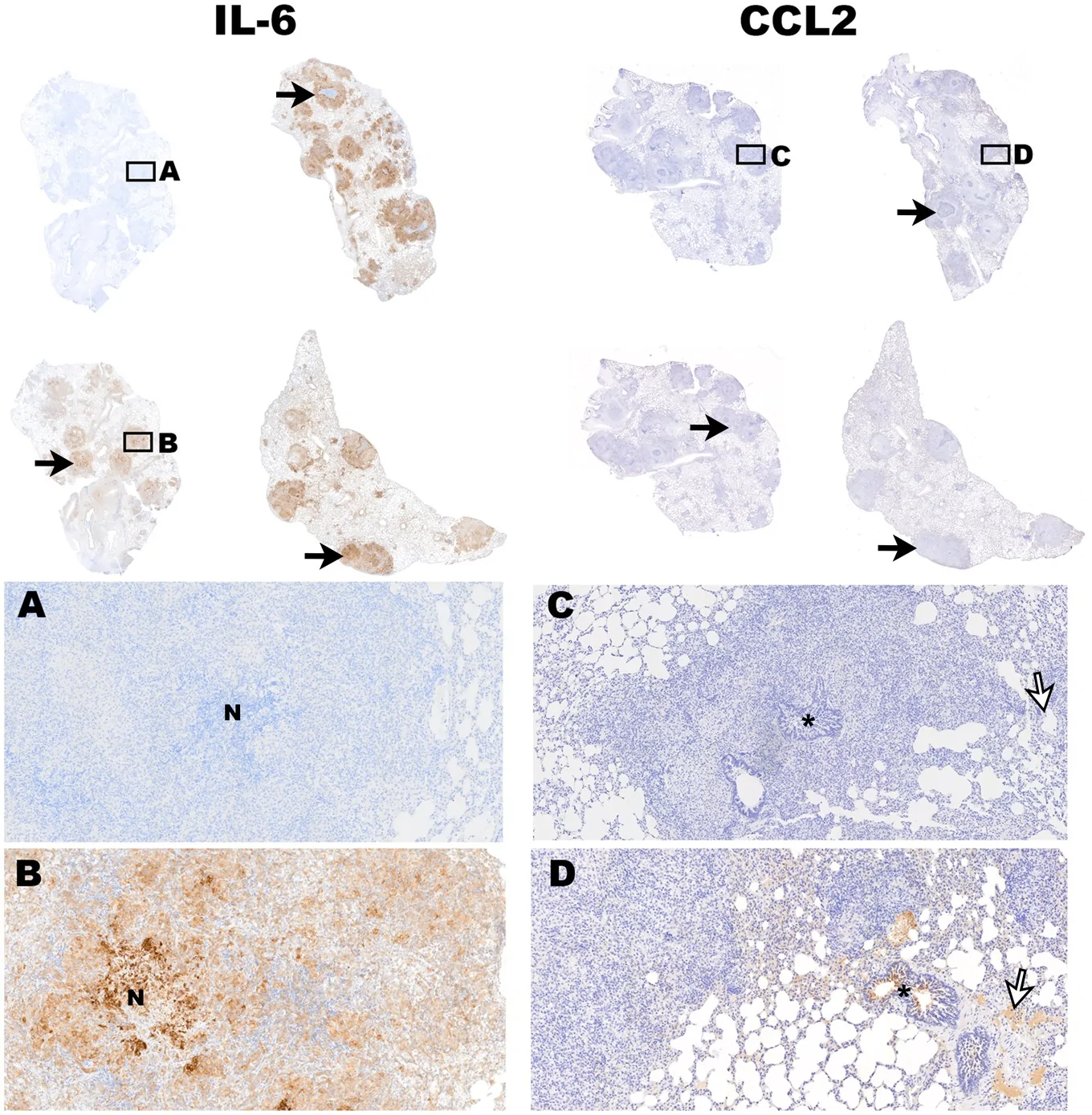
Ex vivo validation using IHC. Lungs from 3 infected guinea pigs were stained for IL-6 (clone 5) and CCL2 (clone 20). IL-6 is shown to be produced by many different cell types across the lung, with higher production concentrated in and around the granulomas. Examples of the multitude of granulomas in each section are indicated by black arrows on the subgross magnification images. The black rectangles indicate the region of a granuloma shown at 10× magnification for the (A) isotype control and (B) anti-guinea pig–IL-6 antibody (clone 5) showing expression in the granuloma wall and in the necrosis (N). CCL2 expression is largely absent from the granulomas, with examples indicated by black arrows. The black rectangles indicate the region of granulomas and adjacent lung tissue shown at 10× magnification for the (C) isotype control and (D) anti-guinea pig–CCL2 antibody (clone 20), where expression is localized to airways (*) and vessels (open arrows).

An in vivo study was conducted with 2 groups: one infected with *Mtb*, using low-dose aerosol, and one uninfected (*n* = 3 guinea pigs per group). Animals were euthanized at 28 days postinfection. Plasma, lung homogenate, and formalin-fixed lung were collected as described. Plasma and lung homogenate were assayed by sandwich ELISA using clone pairs identified as optimal capture and detection clones in the validation process. In the lung homogenate, infected animals produce more TNF-α, IL-6, CCL2, and IL-12p40 compared to their uninfected counterparts. In infected guinea pigs, the average cytokine concentrations in lung were measured at the following concentrations: TNF-α (750 picograms per milliliter-gram of tissue [pg/mL-g]), IL-6 (463 pg/mL-g), IL-12p40 (294 pg/mL-g), and CCL2 (282 pg/mL-g). TNF-α, IL-6, CCL2, and IL-12p40 were also detected in the plasma at lower concentrations (Fig. 6). Of the 4 cytokines quantified using sandwich ELISA, only IL-6 and CCL2 were measurable using IHC (Table S4). IL-6 is seen to be produced by many different cells in the lung, with cells in the granuloma wall producing the most (Fig. 7B). In contrast, CCL2 production could be visualized in the airway epithelium and residual plasma within vessels (Fig. 7D). A rabbit IgG isotype control was incorporated to measure nonspecific binding (Fig. 7A, C).

## Discussion

For the first time, we show thorough evaluation and validation of novel mAbs for use in the guinea pig and as a model of TB. It is often noted that the lack of available immunological reagents for use with the guinea pig is a key limitation to this important in vivo model; however, the data present offer new antibody reagents that will help to overcome this critical limitation.

We evaluated the ability of novel monoclonal recombinant antibodies to detect their intended biological target, both individually in direct ELISA and as a part of a pair in sandwich ELISA. Direct ELISA was chosen as the initial screening tool due to the ability of the assay to evaluate individual antibody reactivity without cross-reactivity from a secondary antibody. Even though clones were shown to be reactive by direct ELISA, not all clones were found to be reactive by sandwich ELISA. Anti-guinea pig–CCL2 antibody clone 19 did not show adequate sensitivity to the intended target by direct ELISA, but when used in sandwich ELISA, as part of a pair, it amplified the sensitivity to the intended target. This highlights that some antibodies may be useful for some, but not all, applications. For IL-2, IL-4, and IL-13, we have determined that antibody clones cannot be used in pair assays. Though it cannot be proven at this time, we hypothesize an issue with steric hindrance where antibodies recognize the same region of the target.^40^ Additionally, antibody pairs for IL-10 and IL-17 were found to only be reactive to the recombinant immunogen, highlighting the need for further optimization using sandwich ELISA.

To validate that the antibodies are specific to the intended target, IP targeting the recombinant immunogen and associated enriched supernatant was conducted. Presence of a band in the supernatant at the same position as recombinant immunogen confirmed successful detection of the biological target by antibodies. IL-2, IL-10, IL-12p40, IL-13, IL-17, and CCL2 were confirmed for specificity using IP. However, not all clones were validated using IP. This could be due to low induction of the specific cytokine, incompatibility with immunoprecipitation reagents, challenges with separation from the precipitating antibody in a high-avidity interaction, or induced conformational changes in antibody or its target.^41^^,^^42^ In an alternative approach, antibody specificity for IL-6 and TNF-α was assessed by morpholino knockdown. Further confirmation of IL-6 specificity was demonstrated by incubating recombinant antigen with the antibody prior to use. Anti-guinea pig–IL-6 antibody clone 5 was confirmed to be specific to the intended target by IHC as there was no visible staining after blocking. All antibodies were trialed for IHC; however, not all clones are suitable for use in IHC. Formalin fixation is well documented to induce epitope conformational changes that interfere with intended antibody specificity.^43^ Accordingly, we also attempted interference at the mRNA level using morpholinos. The reduction in protein translation, resulting from the antisense blocking morpholino interaction, confirms the specificity of the antibody. Morpholino-induced knockdown demonstrated specificity for all targets where antibody pairs functional in ELISA were identified including IL-6, TNF-α, IL-12p40, and CCL2.

Antibody pairs that were deemed to work synergistically in sandwich ELISA were used for ELISpot. Overall, there was a greater number of cytokine-producing cells in stimulated wells compared to unstimulated wells. CCL2 was an exception to this, as it was produced by unstimulated BMDMs. BMDMs that were differentiated from bone marrow progenitor cells using human M-CSF were found to be characterized by the production of CCL2,^44^ which was replicated in the validation of CCL2-targeting antibodies in this study. The use of BMDMs may not be suitable for CCL2 ELISpot. If CCL2-producing cells are required to be quantified, optimizing conditions for quantification using tissue single-cell suspensions (ie lung) would be required.

Innate cytokines have been identified as targets for manipulation by a “hyper-lethal” *Mtb* strain leading to decreased induction of cytokines, such as IL-6, IL-12, and TNF-α.^45^^,^^46^ Select cytokines were located and quantified during *Mtb* infection. Using sandwich ELISA, TNF-α, IL-6, CCL2, and IL-12p40 were measured in the lung and plasma of guinea pigs. Generally, TNF-α was the predominantly produced cytokine among infected guinea pigs when compared to other measured cytokines. However, as expected, there are differences in immune responses between individual guinea pigs.^47^ As determined by IHC, IL-6 was produced by many types of cells in the lung, as expected based on its role during *Mtb* infection.^48^ Observationally, cells in the granuloma produced more IL-6 than any other cell population. CCL2 was produced by epithelial cells lining the airways and is also detected in plasma. The pattern of these cytokine profiles at day 28 post–*Mtb* infection highlighted the well-documented heterogeneity in response to *Mtb* infection in the guinea pig, especially in the context of outbred genetics. These data are consistent with transcriptional profiling of cytokine responses previously performed by laser capture microdissection of granuloma regions in *Mtb*-infected guinea pig lung.^47^ Comparatively, the profile of cytokines measurable by ELISA are similar to commonly used murine models of TB. In the mouse model, early and sustained production of IL-12p40 promotes antigen presentation in lung-draining lymph nodes and activation of cell-mediated immunity.^49^ The induction of IL-6 is required for optimal development of T-cell response, but is not essential for anti-mycobacterial activity in a low-dose aerosol infection.^50^ TNF-α, which dominated in our analysis of guinea pig lung tissue, is essential for typical granuloma structure and phagocyte activation to achieve intracellular control of Mtb growth.^51^^,^^52^ The measurement of these cytokines over the course of infection may be helpful in revealing the kinetic responses that drive the human-like granuloma structure that is characteristic of the guinea pig model.

The tools and methods detailed in this study can aid in the ability to measure and locate the production of innate cytokines and as such will be useful in predicting disease progression. A limitation of this study is centered around the choice of stimuli for supernatant generation. Stimuli used to produce supernatants were originally published in humans or murine models, so stimulatory effects on guinea pig cells were not known. This could have contributed to the inability to fully validate and optimize IL-4 for use in more than one application. Another limitation of this study is the lack of optimization of these antibodies for other single antibody assays, such as flow cytometry. The majority of the cytokine targets outlined here are innate cytokines, which can be further measured in a flow cytometry panel. Additionally, challenges were experienced in generating full-length recombinant immunogens for some cytokines critical in TB pathogenesis and diagnostics. Specifically, IFN-γ was unable to be sufficiently expressed and purified for progression into rabbit immunization. However, mouse mAbs to detect antigen-specific IFN-γ production in guinea pigs have been previously generated and successfully applied in other infectious diseases.^53^^,^^54^ Despite these limitations, this work has validated a novel set of recombinant antibodies that expand the guinea pig–specific reagent repertoire to 9 additional measurable cytokines.

## Supplementary Material

vlag051_Supplementary_Data

## Data Availability

The data underlying this article will be shared on reasonable request to the corresponding author.
