# Supplementary material for: Evaluating novel antibodies for cytokine profiling in the guinea pig model
Source: Immunohorizons. 2026 Sep 22;10(9):vlag051. doi: 10.1093/immhor/vlag051 (PMC13597125; doi:10.1093/immhor/vlag051)
Supplement: Supplementary file 1 [file vlag051_supplementary_data.docx]

**Table S1. Key for antibody clone identification and associated applications**

| **Clone Identifier** | **Company/Vendor** | **Product**  **Information** | **Application** | | | |
| --- | --- | --- | --- | --- | --- | --- |
|  |  |  | **Direct ELISA** | **Sandwich ELISA** | **ELISpot** | **IHC** |
| 1 | QooLabs, Inc. | Rabbit Monoclonal Antibody Anti-Guinea Pig IL-2 **RA0035** | X |  |  |  |
| 2 | QooLabs, Inc. | Rabbit Monoclonal Antibody Anti-Guinea Pig IL-2 **RA0040** | X |  |  |  |
| 3 | QooLabs, Inc. | Rabbit Monoclonal Antibody Anti-Guinea Pig IL-4 **RA0047** | X |  |  |  |
| 4 | QooLabs, Inc. | Rabbit Monoclonal Antibody Anti-Guinea Pig IL-4 **RA0048** | X |  |  |  |
| 5 | QooLabs, Inc. | Rabbit Monoclonal Antibody Anti-Guinea Pig IL-6 **RA0059** | X |  |  | X |
| 6 | QooLabs, Inc. | Rabbit Monoclonal Antibody Anti-Guinea Pig IL-6 **RA0057** | X | X | X |  |
| 7 | QooLabs, Inc. | Rabbit Monoclonal Antibody Anti-Guinea Pig IL-6 **RA0065** | X | X | X |  |
| 8 | QooLabs, Inc. | Rabbit Monoclonal Antibody Anti-Guinea Pig IL-10 **RA0070** | X |  |  | X |
| 9 | QooLabs, Inc. | Rabbit Monoclonal Antibody Anti-Guinea Pig IL-10 **RA0071** | X | X |  |  |
| 10 | QooLabs, Inc. | Rabbit Monoclonal Antibody Anti-Guinea Pig IL-10 **RA0073** | X | X |  |  |
| 11 | QooLabs, Inc. | Rabbit Monoclonal Antibody Anti-Guinea Pig IL-12p40 **RA0075** | X | X | X |  |
| 12 | QooLabs, Inc. | Rabbit Monoclonal Antibody Anti-Guinea Pig IL-12p40 **RA0076** | X | X | X |  |
| 13 | QooLabs, Inc. | Rabbit Monoclonal Antibody Anti-Guinea Pig IL-13 **RA0088** | X |  |  |  |
| 14 | QooLabs, Inc. | Rabbit Monoclonal Antibody Anti-Guinea Pig IL-13 **RA0090** | X |  |  | X |
| 15 | QooLabs, Inc. | Rabbit Monoclonal Antibody Anti-Guinea Pig IL-17 **RA0049** | X | X | X |  |
| 16 | QooLabs, Inc. | Rabbit Monoclonal Antibody Anti-Guinea Pig IL-17 **RA0051** | X | X | X |  |
| 17 | QooLabs, Inc. | Rabbit Monoclonal Antibody Anti-Guinea Pig IL-17 **RA0056** | X |  |  |  |
| 18 | QooLabs, Inc. | Rabbit Monoclonal Antibody Anti-Guinea Pig CCL-2 **GP13-13** | X |  |  |  |
| 19 | QooLabs, Inc. | Rabbit Monoclonal Antibody Anti-Guinea Pig CCL-2 **RA0103** | X | X | X |  |
| 20 | QooLabs, Inc. | Rabbit Monoclonal Antibody Anti-Guinea Pig CCL-2 **RA0105** | X | X | X | X |
| 21 | QooLabs, Inc. | Rabbit Monoclonal Antibody Anti-Guinea Pig TNFα **RA0095** | X | X | X |  |
| 22 | QooLabs, Inc. | Rabbit Monoclonal Antibody Anti-Guinea Pig TNFα **RA0391** | X | X | X |  |

**Table S2. Summary of raw absorbance values (OD_450_) used in normalization of direct ELISA data to evaluate reactivity among stimulated and unstimulated supernatant (SN).**

**Figure S1. Antibody specificity demonstrated by detection of respective immunogens for each antibody clone.** Recombinant immunogens were detected at a concentration of 100 ng/ml by direct ELISA (x symbol), compared to media-only negative control (open circles).

**Figure S2. Validation of antibody specificity using immunoprecipitation.** Red boxes indicate bands of specific target immunoprecipitated from enriched supernatant.

**Table S3. Targets and sequences of morpholinos used for confirmation of antibody specificity.**  The translation-blocking morpholino oligo sequences are provided from 5' to 3' and complementary to each translation-blocking target.

| Target | Ensembl (ENS) or NCBI Reference | Morpholino sequence |
| --- | --- | --- |
| TNFα | ENSCPOG00000002343.4 | 5'-CCCGGATCATGCTTTCTGTGCTCAT |
| CCL2 | ENSCPOG00000013468.4 | 5'-CTTCTGTGCTCTTCTGGTAGCTCTG |
| IL-12p40 | ENSCPOG00000001622.4 | 5'-AGGAGATCAACTGCCTGTGACACAT |
| IL-6 | XM_013152399.3 | 5'-TCGTTGTGGAGAGGAACTTCAT |

**Table S4. Optimized methods for clones functional by IHC on formalin-fixed paraffin-embedded tissue.**

| **Target** | **Clone** | **Dilution** | **Antigen Retrieval** |
| --- | --- | --- | --- |
| CCL-2 | RA0105 | 1:100 | ER2 20 minutes |
| IL-6 | RA0059 | 1:100 | ER1 40 minutes |
| IL-13 | RA0090 | 1:100 | ER1 40 minutes |
| IL-10 | RA0070 | 1:100 | ER2 20 minutes |

**Figure S3. Specificity of IL-6 clone 5 demonstrated by blocked IHC.** Lung from two *Mtb* infected guinea pigs are shown in left and right columns. Recombinant IL-6 immunogen was incubated with anti-IL-6 clone 5 in equimolar concentration prior to application and the primary antibody complex on infected lung tissue. Pre-incubation with recombinant IL-6 abrogated immunoreactivity as shown in subgross images (A) and granulomas at 5X magnification (B). IL-6 was readily detected by clone 5 in the absence of recombinant IL-6 immunogen in granuloma regions of infected lung, as in subgross (C) and granuloma regions at 5X magnification (D).
